# Evolutionary dynamics of health food safety regulatory information disclosure from the perspective of consumer participation

**DOI:** 10.1002/fsn3.1257

**Published:** 2019-11-22

**Authors:** Jun Luo, Tingqiang Chen, Jinnan Pan

**Affiliations:** ^1^ School of Health Economics and Management Nanjing University of Chinese Medicine Nanjing China; ^2^ School of Economics and Management Nanjing Tech University Nanjing China

**Keywords:** consumer participation, evolution model, health food, regulatory information disclosure, safety risk

## Abstract

The “trust” attribute of health food has intensified the serious information asymmetry problem in the health food market. Moreover, it has strengthened the formation and influence of potential or hidden safety risks of health food, bringing huge impacts on social harmony and stability. Therefore, this study builds a model of health food enterprise production and operation and government regulatory information disclosure from the perspective of consumer participation. The study fully considers the government's policy burdens and the degree of consumer response to food safety regulatory information. Moreover, it aims to explore the evolution mechanism of food enterprise production and operation strategies and government regulatory information disclosure strategies and analyze the evolution process of the government behavior in health food safety regulatory information disclosure. The theoretical derivation and simulation analysis found that the more sensitive consumers are to health food safety regulatory information, the more significant the profitability improvement of enterprises producing quality health food, thus urging the government to make an objective and comprehensive disclosure of health food safety regulatory information.

## Graphical abstract

This study is significant in supervising and managing health food safety because it analyzes the evolution process of the government behavior in health food safety regulatory information disclosure from the perspective of consumer participation.

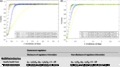

## 1 INTRODUCTION

Health food is a special kind of food that have a specific health functions (Franco, Diez Roux, Glass, Caballero, & Brancati, 2008; Story, Kaphingst, Robinson‐O'Brien, & Glanz, 2008). “Trust” and “experience” are the typical attributes of health food. Health food enterprises often use these attributes for illegal production to produce excess profits, resulting in evident or potential safety risks. The direct factors that form health food safety risks are the illegal production and operation of health food enterprises, which are mainly driven by personal interests. First, regulators and consumers cannot often make timely and accurate judgment on the real quality of health food due to the said attributes of health food (Darby & Karni, 1973; Pu, Lu, & Han, 2014). Illegal production and operations of health food enterprises have less recognition probability, which leads to serious “adverse selection” in the health food market. Second, as the quality of health food is difficult to distinguish, the willingness to pay of health food consumers is often low. In addition, the cost paid by health food enterprises for the production of high‐quality health food is difficult to make up for and may be exacerbated by defective health food (Dulleck, Kerschbamer, & Sutter, 2009). As a result, “bad money drives out good money” (Akerlof, 1970). Finally, information asymmetry in the health food market provides convenience for the generation and diffusion of health food safety risks, which is also a key cause for the frequent occurrence of health food safety risks (Chen, Ma, & Wang, 2018; Wang, Chen, & Wang, 2015). The information asymmetry problem in the health food market happens between the upstream and downstream enterprises of health food, between health food enterprises and government regulators, and between health food enterprises and health food consumers. Among them, the information asymmetry problem between the latter is the most serious (Waldman & Kerr, 2018; Wang & Chen, 2016). In addition, the “trust” attribute of health food gives health food consumers, which are already at an information disadvantage, a difficult time to make an accurate judgment on food quality. This situation certainly causes health food safety risks.

In recent years, the prevention and control of food safety risks to guarantee the “safety on the tip of one's tongue” remain the focus of theoretical and practical research. Regarding the safety risk management under the influence of the “trust” attribute of health food, some scholars have proposed that governments need to strengthen administrative and judicial discipline to ensure health food safety (Ni & Zeng, 2009), establish an internationally accepted certification system (Bai, Ma, Yang, Zhao, & Gong, 2007; Fan et al., 2009), and improve information transparency, among others (Chen, Wang, & Wang, 2017; Nyokabi et al., 2017). Food safety certification has attracted worldwide attention. For instance, Teixeira and Sampaio (2013) studied nationwide research projects related to ISO 22000 in Portugal. They found a pioneering contribution in order to study food safety management systems adoption by Portuguese companies. Milios, Zoiopoulos, Pantouvakis, Mataragas, and Drosinos (2013) evaluated the food safety management system (HACCP‐type system) implemented in Greek food businesses, examined the techno‐managerial factors influencing its application according to enterprises' opinion and correlate these answers to the HACCP evaluation results. In the past few years, in Romania, a number of companies began to certify food safety management systems according to ISO 22000, IFS or BRC standards. The common ground is the HACCP study, which is included in all food safety standards (Chira, Chira, & Delian, 2011). However, food safety management mentioned above is all about the technology of enterprises, not the government or consumers. Moreover, Powell et al. (2013) put forward that audits and inspections are never enough. Based on the establishment of a strong food safety culture throughout the food safety system, they identified the limitations of food safety inspections and audits and proposed suggestions for strengthening the system. Fernando, Ng, and Walters (2015) investigated the contribution of regulatory incentives offered by regulators as a moderator variable enhancing adoption of Malaysian food safety system (MeSTI). They took regulators into account but no participation of consumers. At the same time, in the process of health food safety governance, the interaction of factors, such as information opacity and weak social supervision, leads to poor supervision effect. As a result, health food safety risk incidents often occur. The integration of all social forces to participate in health food safety governance is thus necessary (Li, Bernard, Johnston, Messer, & Kaiser, 2017; Martinez, Verbruggen, & Fearne, 2013). Consumers in the health food market can have sufficient information to determine the quality of health food and government regulators can efficiently perform regulatory activities only by giving full play to social forces (Arthur, 2014; Rosenau & Czempiel, 1992). Such processes effectively reduce the occurrence probability and frequency of health food safety risks. Existing studies have shown that the main reason for the frequent occurrence of health food safety problems is the serious information asymmetry problem among stakeholders in the food supply network (Boxstael et al., 2013; Wang & Chen, 2016; Wanget al., 2015). Studies have also shown that 82 percent of consumers need health food safety information services (Wilsonet, Chapman, & Powell, 2011). This finding reflects the serious information asymmetry problem between consumers and health food enterprises, severely hindering the process of consumer participation in health food safety social governance. To effectively solve the information asymmetry problem in the health food market and thus promote the healthy development of this market, domestic and foreign scholars have conducted beneficial explorations on this issue, such as analyzing and discussing the subjects and obligations of health food safety information disclosure from the angle of legal theory (Firdauset, Son, & Mohhiddin, 2015). Among the many subjects of health food safety information disclosure, government regulatory departments hold all health food safety information in their supervision process, and such information disclosure can help consumers to accurately judge the quality of health food and lay a foundation for consumers to “vote with their feet” (König, 2010; Wu, 2012). Furthermore, from the perspective of social development, the public's demand for the “right to know” increasingly becomes high (Kleve & Mulder, 2008; Sawadaet, Kondo, & Ito, 2009). The government's disclosure of information has also become a popular topic in the society (Liu, Chen, & Su, 2013; Umehara & Ohta, 2009; Wang, 2015). In reality, regulatory capture caused by policy burden, as well as the serious incentive distortion in the current supervision system of “result evaluation system” and “the combination of the right of detection and the right of punishment,” can make regulators and health food enterprises collude and conceal health food safety information (Dreyer, Renn, Cope, & Frewer, 2010; Helm, 2006; Zimper & Hassan, 2012). At the same time, considering that the health food market is a typical trust product market, the occurrence of health food safety accidents may lead to two effects, that is, contagion and competitive effects (Wang, Liu, & Du, 2014). The microscopic manifestation of the two effects lies in the adjustment of consumers’ consumption behavior in accordance with health food safety information. The two different effects directly affect the earnings and thus the strategic choice of health food enterprises and government regulators. In fact, in a market with competitive effect, consumer participation has a significant impact on the government regulatory authority health food safety regulatory information disclosure strategies and health food enterprise production strategies.

To sum up, the “trust” attribute of health food aggravates the serious information asymmetry problem in the health food market. This attribute also intensifies the formation and influence of potential or hidden safety risk of health food, bringing huge impacts on social harmony and stability. However, at present, no relevant research has analyzed and discussed the influencing mechanisms of consumer responses to food safety regulatory information on government regulatory information disclosure strategies and food enterprise production and operation strategies. Therefore, this study builds a model of health food enterprise production and operation and government regulatory information disclosure from the perspective of consumer participation. It fully considers the government's policy burdens and the degree of consumer responses to food safety regulatory information. Moreover, this study aims to explore the evolution mechanism of food enterprise production and operation strategies and government regulatory information disclosure strategies. The findings of this study are expected to promote a healthy development of the food market and ensure “safety on the tip of one's tongue.”

## 2 MODEL CONSTRUCTION

### 2.1 Model assumption

Assumption 1 Under the influence of policy burden and regulatory capture, the phenomenon that governments collude with health food enterprises exists. That is, governments selectively disclose health food safety supervision information obtained in the supervision process to reduce the impacts of negative news on health food enterprises. The choice of government health food safety supervision information disclosure strategy will also be affected by other factors, such as consumer response to information and social credibility. For health food enterprises, the selection of production and operation strategies can be adjusted according to the government's information disclosure behavior and consumers’ sensitivity to health food safety supervision information. Based on the analysis above, the strategic space of government regulators can be expressed as *S_G_ = {Disclosure of regulatory information, Nondisclosure of regulatory information}*. In addition, the strategic space of health food enterprises can be expressed as *S_c_ = {Production and operation of quality health food, Production and operation of inferior health food}.*

Assumption 2 Government benefits consist of two parts, one is the policy burden borne by health food enterprises and the other is consumer utility, which can be expressed as $R_{G}=\lambda R_{C}+\left(1-\lambda \right)R_{A}$. Among them, λ represents the policy burden that an enterprise needs to bear, *R_c_* represents the benefits of health food enterprises, and *R_A_* represents the utility that consumers acquire from buying health food. According to the demand function in economics, the benefits of health food enterprises can be concretized into $R_{C}=\left(P-C\right)Q$. Among them, Q=a-bP, where *P* represents the price, *Q* represents the quantity demand, *C* represents the cost, *a* is the market demand for the health food in period *t* − 1, and *b* is the effect of unit price change on the quantity demand. The utility that consumers can obtain by purchasing health food mainly depends on the quality of health food. Therefore, the utility function of consumers can be assumed as follows:

(1) $$R_{A}=\left\{\begin{array}{c} B,\;\text{if buy quality health food}\\ b,\;\text{if buy inferior health food} \end{array}\right..$$

Assumption 3 Consumers have heterogeneity in the ability to handle regulatory information, which is directly reflected in their sensitivity to such information. Consumers’ sensitivity to health food safety regulatory information is reflected in this model, which has a main effect on enterprise income. Consumers have strong information processing ability. Thus, they are highly sensitive to health food safety supervision information. If health food enterprises produce quality health food, then the disclosure of supervision information can strongly affect consumers’ purchasing behavior, thereby improving the earnings of health food enterprises. We represent consumers’ sensitivity to regulatory information as $\delta_{h}$($\delta_{h}\geq 1$). On the contrary, if health food enterprises produce inferior health food, then the disclosure of regulatory information can weaken the effect on consumers’ purchasing behavior, thereby reducing the income of health food enterprises. The impact on the earnings of enterprises producing inferior health food is expressed as $\delta_{l}$($\delta_{l}\leq 1$).

Assumption 4 When health food enterprises produce and sell inferior health food in the supervision process, they must assume the fine *F*. If enterprises want to form collusion with the government, they need to bear regulatory capture cost *C_b_*. If the government finds inferior health food in the supervision process and fails to give timely warning, then the occurrence of health food safety accidents can cause losses to its social credibility, and the loss value is *R*.

### 2.2 Model building

On the basis of the assumptions in Section 2.1 and the actual operation of the health food market, the benefit matrix of health food enterprise and government regulator health food safety risk management can be attained, as shown in Table 1 refer to appendix. Table 1 shows that the expected benefits of the government's decision to disclose health food safety supervision information *E*_G1_ are as follows:

(2) $$E_{G1}=\mu \left[\lambda \left(p_{h}-c_{h}\right)\delta_{h}Q_{\mathrm{th}}+\left(1-\lambda \right)B\right]+\left(1-\mu \right)\left[\lambda \left(p_{l}-c_{l}\right)\delta_{l}Q_{\mathrm{tl}}+\left(1-\lambda \right)b+F\right].$$

**Table 1:** Benefit matrix of health food safety risk management

	Government regulators	
	Disclosure of regulatory information	Non‐disclosure of regulatory information
Health food enterprises		
Quality health food	$\left(p_{h}-c_{h}\right)\delta_{h}Q_{\mathrm{th}}$, $\lambda \left(p_{h}-c_{h}\right)\delta_{h}Q_{\mathrm{th}}+\left(1-\lambda \right)B$	$\left(p_{h}-c_{h}\right)Q_{\mathrm{th}}$, $\lambda \left(p_{h}-c_{h}\right)Q_{\mathrm{th}}+\left(1-\lambda \right)B$
Inferior health food	$\left(p_{l}-c_{l}\right)\delta_{l}Q_{\mathrm{tl}}-F$, $\lambda \left(p_{l}-c_{l}\right)\delta_{l}Q_{\mathrm{tl}}+\left(1-\lambda \right)b+F$	$\left(p_{l}-c_{l}\right)Q_{\mathrm{tl}}-F-C_{b}$, $\lambda \left(p_{l}-c_{l}\right)Q_{\mathrm{tl}}+\left(1-\lambda \right)b+F+C_{b}-R$

The expected benefits of the government's choice of concealing health food safety regulatory information *E*_G2_ are as follows:

(3) $$E_{G2}=\mu \left[\lambda \left(p_{h}-c_{h}\right)Q_{\mathrm{th}}+\left(1-\lambda \right)B\right]+\left(1-\mu \right)\left[\lambda \left(p_{l}-c_{l}\right)Q_{\mathrm{tl}}+\left(1-\lambda \right)b+F+C_{b}-R\right].$$

Therefore, the average benefits *E_G_* of the behavioral strategy selection of the government supervision department for health food safety risk supervision information are obtained:

(4) $${\bar{E}}_{G}=qE_{G1}+\left(1-q\right)E_{G2}.$$

Similarly, the expected benefits *E_C_*_1_ of health food enterprises in producing quality health food, the expected benefits *E_C_*_2_ of producing inferior quality health food, and the average benefits ${\bar{E}}_{C}$ of behavior strategy selection of health food enterprises for health food safety risk supervision information are, respectively, as follows:

(5) $$E_{C1}=q\left(p_{h}-c_{h}\right)\delta_{h}Q_{\mathrm{th}}+\left(1-q\right)\left(p_{h}-c_{h}\right)Q_{\mathrm{th}}.$$

(6) $$E_{C2}=q\left[\left(p_{l}-c_{l}\right)\delta_{l}Q_{\mathrm{tl}}-F\right]+\left(1-q\right)\left[\left(p_{l}-c_{l}\right)Q_{\mathrm{tl}}-F-C_{b}\right].$$

(7) $${\bar{E}}_{C}=\mu E_{C1}+\left(1-\mu \right)E_{C2}.$$

The dynamic replication equation of evolutionary game theory can be obtained from the perspective of consumer participation to the government's supervision of health food safety risks and health food enterprise selection of behavioral strategy:

(8) $$\frac{d\mu }{dt}=\mu \left(1-\mu \right)\left\{\left[q\delta_{h}+\left(1-q\right)\right]\left(p_{h}-c_{h}\right)Q_{\mathrm{th}}-\left[q\delta_{l}+\left(1-q\right)\right]\left(p_{l}-c_{l}\right)Q_{\mathrm{tl}}+F+\left(1-q\right)C_{b}\right\}.$$

(9) $$\frac{dq}{dt}=q\left(1-q\right)\left[\mu \lambda Q_{\mathrm{th}}\left(p_{h}-c_{h}\right)\left(\delta_{h}-1\right)+\left(1-\mu \right)\lambda Q_{\mathrm{tl}}\left(p_{l}-c_{l}\right)\left(\delta_{l}-1\right)+\left(1-\mu \right)\left(R-C_{b}\right)\right].$$

Therefore, the evolutionary game model of health food safety risk supervision from the perspective of consumer participation is formed by Equations (8) and (9). By analyzing the evolutionary game model, we can describe and interpret the influence factors and evolutionary dynamic characteristics of behavior strategy choice of government regulators and health food enterprises in health food safety risk regulation.

## 3 MODEL THEORETICAL ANALYSIS

According to the game differential equation dynamic system of health food safety risk supervision evolution from the perspective of consumer participation formed by Equations (8) and (9), the evolution process of the strategy of government supervision departments and health food enterprises can be described well. Combined with the solving process of the evolutionary game, five equilibrium points of the evolutionary system can be obtained: *E*_1_(0, 0), *E*_2_ (0, 1), *E*_3_ (1, 0), *E*_4_ (1, 1), and ($\mu^{*}$, $q^{*}$), two of which are shown as follows:

(10) $$q^{*}=-\frac{\left(p_{h}-c_{h}\right)Q_{\mathrm{th}}-\left(p_{l}-c_{l}\right)Q_{\mathrm{tl}}-C_{b}}{\left(\delta_{h}-1\right)\left(p_{h}-c_{h}\right)Q_{\mathrm{th}}-\left(\delta_{l}-1\right)\left(p_{l}-c_{l}\right)Q_{\mathrm{tl}}+F-C_{b}}.$$

(11) $$\mu^{*}=-\frac{\lambda Q_{\mathrm{tl}}\left(\delta_{l}-1\right)\left(p_{l}-c_{l}\right)+R-C_{b}}{\lambda Q_{\mathrm{th}}\left(\delta_{h}-1\right)\left(p_{h}-c_{h}\right)-\lambda Q_{\mathrm{tl}}\left(\delta_{l}-1\right)\left(p_{l}-c_{l}\right)-\left(R-C_{b}\right)}.$$

In the dynamic evolving system, the five equilibrium points are not necessarily an evolutionary stable strategy (ESS) of the evolutionary game system of health food safety risk supervision from the perspective of consumer participation. According to the *Friedman* method, *Jacobian* matrix is constructed to determine the local stability of the equilibrium points in the dynamic evolving system. We can obtain the *Jacobian* matrix from the evolutionary game dynamic system of health food safety risk supervision from the perspective of consumer participation formed by Equations (8) and (9):

(12) $$J=\left[\begin{array}{cc} \frac{\partial \dot{\mu }}{\partial \mu } & \frac{\partial \dot{\mu }}{\partial q}\\ \frac{\partial \dot{q}}{\partial \mu } & \frac{\partial \dot{q}}{\partial q} \end{array}\right]=\left[\begin{array}{cc} a_{11} & a_{12}\\ a_{21} & a_{22} \end{array}\right].$$

Among them, the values of the elements in the *Jacobian* matrix are as follows:

$$a_{11}=\left(1-2\mu \right)\left\{\left[q\delta_{h}+\left(1-q\right)\right]\left(p_{h}-c_{h}\right)Q_{\mathrm{th}}-\left[q\delta_{l}+\left(1-q\right)\right]\left(p_{l}-c_{l}\right)Q_{\mathrm{tl}}+F+\left(1-q\right)C_{b}\right\}.$$

$$a_{12}=\mu \left(1-\mu \right)\left[\left(\delta_{h}-1\right)\left(p_{h}-c_{h}\right)Q_{\mathrm{th}}-\left(\delta_{l}-1\right)\left(p_{l}-c_{l}\right)Q_{\mathrm{tl}}-C_{b}\right].$$

$$a_{21}=q\left(1-q\right)\left[\lambda \left(\delta_{h}-1\right)\left(p_{h}-c_{h}\right)Q_{\mathrm{th}}-\lambda \left(p_{l}-c_{l}\right)Q_{\mathrm{tl}}-\left(F-C_{b}+R\right)\right].$$

$$a_{22}=\left(1-2q\right)\left[\mu \lambda Q_{\mathrm{th}}\left(p_{h}-c_{h}\right)\left(\delta_{h}-1\right)+\left(1-\mu \right)\lambda Q_{\mathrm{tl}}\left(p_{l}-c_{l}\right)\left(\delta_{l}-1\right)+\left(1-\mu \right)\left(R-C_{b}\right)\right].$$

According to the given evolutionary game dynamic system, we can obtain the specific values of the five equilibrium points at $a_{11}$, $a_{12}$, $a_{21}$, and $a_{22}$, as shown in Table 2 refer to appendix. In Table 2, Λ and Γ are respectively expressed as

(13) $$\Lambda =-\frac{\lambda Q_{\mathrm{th}}\left(\delta_{h}-1\right)\left(p_{h}-c_{h}\right)\left[\lambda Q_{\mathrm{tl}}\left(\delta_{l}-1\right)\left(p_{l}-c_{l}\right)+F+R-C_{b}\right]\left[\left(\delta_{h}-1\right)\left(p_{h}-c_{h}\right)Q_{\mathrm{th}}-\left(\delta_{l}-1\right)\left(p_{l}-c_{l}\right)Q_{\mathrm{tl}}+F-C_{b}\right]}{{\left[\lambda Q_{\mathrm{th}}\left(\delta_{h}-1\right)\left(p_{h}-c_{h}\right)-\lambda Q_{\mathrm{tl}}\left(\delta_{l}-1\right)\left(p_{l}-c_{l}\right)-\left(F+R-C_{b}\right)\right]}^{2}}.$$

(14) $$\Gamma =-\frac{\left[\delta_{h}\left(p_{h}-c_{h}\right)Q_{\mathrm{th}}-\delta_{l}\left(p_{l}-c_{l}\right)Q_{\mathrm{tl}}+F\right]\left[\left(p_{h}-c_{h}\right)Q_{\mathrm{th}}-\left(p_{l}-c_{l}\right)Q_{\mathrm{tl}}-C_{b}\right]}{{\left[\left(\delta_{h}-1\right)\left(p_{h}-c_{h}\right)Q_{\mathrm{th}}-\left(\delta_{l}-1\right)\left(p_{l}-c_{l}\right)Q_{\mathrm{tl}}+F+C_{b}\right]}^{2}}\left[\lambda \left(\delta_{h}-1\right)\left(p_{h}-c_{h}\right)Q_{\mathrm{th}}-\lambda \left(p_{l}-c_{l}\right)Q_{\mathrm{tl}}-\left(F-C_{b}+R\right)\right].$$

**Table 2:** Specific values of local equilibrium points at $a_{11}$, $a_{12}$, $a_{21}$, and $a_{22}$

Equilibrium points	$a_{11}$	$a_{12}$	$a_{21}$	$a_{22}$
(0, 0)	$\left(p_{h}-c_{h}\right)Q_{\mathrm{th}}-\left(p_{l}-c_{l}\right)Q_{\mathrm{tl}}+F+C_{b}$	0	0	$\lambda Q_{\mathrm{tl}}\left(p_{l}-c_{l}\right)\left(\delta_{l}-1\right)+R-C_{b}$
(0, 1)	$\delta_{h}\left(p_{h}-c_{h}\right)Q_{\mathrm{th}}-\delta_{l}\left(p_{l}-c_{l}\right)Q_{\mathrm{tl}}+F$	0	0	$-[\lambda Q_{\mathrm{tl}}\left(p_{l}-c_{l}\right)\left(\delta_{l}-1\right)+R-C_{b}]$
(1, 0)	$-\left(p_{h}-c_{h}\right)Q_{\mathrm{th}}+\left(p_{l}-c_{l}\right)Q_{\mathrm{tl}}-F-C_{b}$	0	0	$\lambda Q_{\mathrm{th}}\left(p_{h}-c_{h}\right)\left(\delta_{h}-1\right)$
(1, 1)	$-\delta_{h}\left(p_{h}-c_{h}\right)Q_{\mathrm{th}}+\delta_{l}\left(p_{l}-c_{l}\right)Q_{\mathrm{tl}}-F$	0	0	$-\lambda Q_{\mathrm{th}}\left(p_{h}-c_{h}\right)\left(\delta_{h}-1\right)$
($\mu^{*}$, $q^{*}$)	0	Λ	Γ	0

Theorem 1 When $\left(p_{h}-c_{h}\right)Q_{\mathrm{th}}-\left(p_{l}-c_{l}\right)Q_{\mathrm{tl}}>0$, the evolutionary game system of health food safety risk supervision from the perspective of consumer participation has the evolutionary stability strategy ESS (1, 1).

Without considering the effects of regulatory information disclosure but with a deepening understanding of the government and health food enterprises on the sensitivity of regulatory information for consumers, if the net income of enterprises in the production of high‐quality health food is greater than that in the production of inferior health food, then the evolutionary game result of health food safety risk supervision from the perspective of consumer participation can converge to equilibrium *E*_4 _(1, 1). That is, when consumers have sufficient identification ability on the quality of health food, they can proactively decide to buy quality health food and expel enterprises producing poor‐quality health food through market means. This ability is possible even if the government does not disclose regulatory information. In addition, health food enterprises can obtain high profits through the production of quality health food. Therefore, the production of quality health food is an optimal decision of health food enterprises. From the perspective of the government, if governments do not voluntarily disclose health food safety supervision information, then the public credibility of the government can be affected if health food safety problems occur. Thus, government regulatory departments must make health food safety regulatory information available to the public and improve their transparency.

Theorem 2 When $\left(p_{h}-c_{h}\right)Q_{\mathrm{th}}-\left[\left(p_{l}-c_{l}\right)Q_{\mathrm{tl}}-F-C_{b}\right]<0$, $\delta_{h}\left(p_{h}-c_{h}\right)Q_{\mathrm{th}}-\left[\delta_{l}\left(p_{l}-c_{l}\right)Q_{\mathrm{tl}}-F\right]>0$, and $\lambda \delta_{l}\left(p_{l}-c_{l}\right)Q_{\mathrm{tl}}-\left[\lambda \left(p_{l}-c_{l}\right)Q_{\mathrm{tl}}+C_{b}-R\right]>0$, the evolutionary game system of health food safety risk supervision from the perspective of consumer participation has the evolutionary stability strategy ESS (1, 1).

When consumers are sensitive to health food safety risk regulatory information, that is, consumers can timely adjust their purchase strategy in accordance with the public health food safety regulatory information of government regulators, health food safety risk regulatory information disclosure can improve the profitability of health food manufacturing enterprises, which produce quality health food. In this situation, if the government's benefits from regulatory information disclosure are no more than the benefits from regulatory information nondisclosure, then the evolutionary game result of health food safety risk supervision from the perspective of consumer participation can converge to equilibrium *E*_4_ (1, 1). Constraint conditions $\left(p_{h}-c_{h}\right)Q_{\mathrm{th}}-\left[\left(p_{l}-c_{l}\right)Q_{\mathrm{tl}}-F-C_{b}\right]<0$, $\delta_{h}\left(p_{h}-c_{h}\right)Q_{\mathrm{th}}-\left[\delta_{l}\left(p_{l}-c_{l}\right)Q_{\mathrm{tl}}-F\right]>0$, and $\lambda \delta_{l}\left(p_{l}-c_{l}\right)Q_{\mathrm{tl}}-\left[\lambda \left(p_{l}-c_{l}\right)Q_{\mathrm{tl}}+C_{b}-R\right]>0$ mean that the disclosure of health food safety supervision information can significantly improve the profitability of enterprises producing quality health food as consumers do not have sufficient capacity to identify the quality of health food. Therefore, under the influence of government regulatory information disclosure on the enhancement of the purchasing behavior of consumers, health food enterprises can produce quality health food as they learn from the behaviors of government regulatory departments and consumers. In addition, for government regulators, the disclosure of regulatory information disclosure, as an important means to improve the transparency of regulatory information on health food safety, can improve the net profits of enterprises producing high‐quality health food. Thus, the government's earnings accordingly increase. Moreover, as the profits gained by collusion cannot make up for the loss caused by the declining social credibility, the government must publicize health food safety supervision information and improve its transparency.

Theorem 3 When $\left(p_{h}-c_{h}\right)Q_{\mathrm{th}}-\left[\left(p_{l}-c_{l}\right)Q_{\mathrm{tl}}-F-C_{b}\right]<0$, $\delta_{h}\left(p_{h}-c_{h}\right)Q_{\mathrm{th}}-\left[\delta_{l}\left(p_{l}-c_{l}\right)Q_{\mathrm{tl}}-F\right]<0$, and $\lambda \delta_{l}\left(p_{l}-c_{l}\right)Q_{\mathrm{tl}}-\left[\lambda \left(p_{l}-c_{l}\right)Q_{\mathrm{tl}}+C_{b}-R\right]>0$, the evolutionary game system of health food safety risk supervision from the perspective of consumers participation has the evolutionary stability strategy ESS (0, 1).

For instance, in the process of consumption, consumers cannot effectively use the regulatory information disclosed by government regulators. In this case, when consumers are not sensitive to health food safety risk regulatory information, significantly improving the profitability of enterprises producing quality health food with the enhanced effect of government regulatory information disclosure on the purchasing behavior of consumers is difficult. The willingness of health food enterprises to produce quality health food is insufficient, and the possibility of continuing to produce inferior health food is high. The benefits obtained by government regulators from conspiring with health food enterprises cannot compensate for the loss of social credibility. The benefits gained from disclosing regulatory information are higher than those gained from not disclosing such information. Eventually, the evolutionary game result of health food safety risk supervision from the perspective of consumer participation can converge to equilibrium $E_{2}$ (0,1). In other words, when the disclosure of government regulatory information fails to effectively improve the profitability of enterprises producing quality health food, the willingness of health food enterprises to produce quality health food is insufficient. Considering the impact of public credibility, disclosing government supervision information on health food safety and guiding consumers to buy quality health food will be the main strategies of government supervision departments.

Theorem 4 When $\left(p_{h}-c_{h}\right)Q_{\mathrm{th}}-\left[\left(p_{l}-c_{l}\right)Q_{\mathrm{tl}}-F-C_{b}\right]<0$, $\delta_{h}\left(p_{h}-c_{h}\right)Q_{\mathrm{th}}-\left[\delta_{l}\left(p_{l}-c_{l}\right)Q_{\mathrm{tl}}-F\right]<0$, and $\lambda \delta_{l}\left(p_{l}-c_{l}\right)Q_{\mathrm{tl}}-\left[\lambda \left(p_{l}-c_{l}\right)Q_{\mathrm{tl}}+C_{b}-R\right]<0$, the evolutionary game system of health food safety risk supervision from the perspective of consumer participation has the evolutionary stability strategy ESS(0,0).

When $\left(p_{h}-c_{h}\right)Q_{\mathrm{th}}-\left[\left(p_{l}-c_{l}\right)Q_{\mathrm{tl}}-F-C_{b}\right]<0$,$\delta_{h}\left(p_{h}-c_{h}\right)Q_{\mathrm{th}}-\left[\delta_{l}\left(p_{l}-c_{l}\right)Q_{\mathrm{tl}}-F\right]<0$, and $\lambda \delta_{l}\left(p_{l}-c_{l}\right)Q_{\mathrm{tl}}-\left[\lambda \left(p_{l}-c_{l}\right)Q_{\mathrm{tl}}+C_{b}-R\right]<0$, that is, consumers are not sensitive to health food safety risk regulatory information, the disclosure of government regulatory information cannot effectively improve the profitability of enterprises producing quality health food. Moreover, if the government does not pay enough attention to reputation loss, then the evolutionary game result of health food safety risk supervision from the perspective of consumer participation can converge to equilibrium *E*_1_ (0, 0). That is, when consumers are not sensitive to the supervision information on health food safety and when their ability of identifying the quality of health food is insufficient, the profitability of health food enterprises producing quality health food will be lower than that of health food enterprises producing poor‐quality health food. Therefore, in the health food market, the willingness of health food enterprises to produce quality health food is insufficient, and the possibility of producing poor‐quality health food gradually increases. In addition, if government regulators do not pay enough attention to public credibility, then they will choose not to disclose regulatory information on health food safety when considering the policy burden.

## 4 COMPUTATIONAL EXPERIMENT AND SIMULATION

Computer simulation analysis is the most effective way to test real‐time dynamic data without a large number of empirical validations. Hence, the software MATLAB 2016b is used to simulate the evolutionary dynamics of health food safety risk supervision from the perspective of consumer participation.

### 4.1 Simulation of the evolutionary dynamics of health food safety risk supervision when $\left(p_{h}-c_{h}\right)Q_{\mathrm{th}}-\left(p_{l}-c_{l}\right)Q_{\mathrm{tl}}>0$

**Figure 1:**
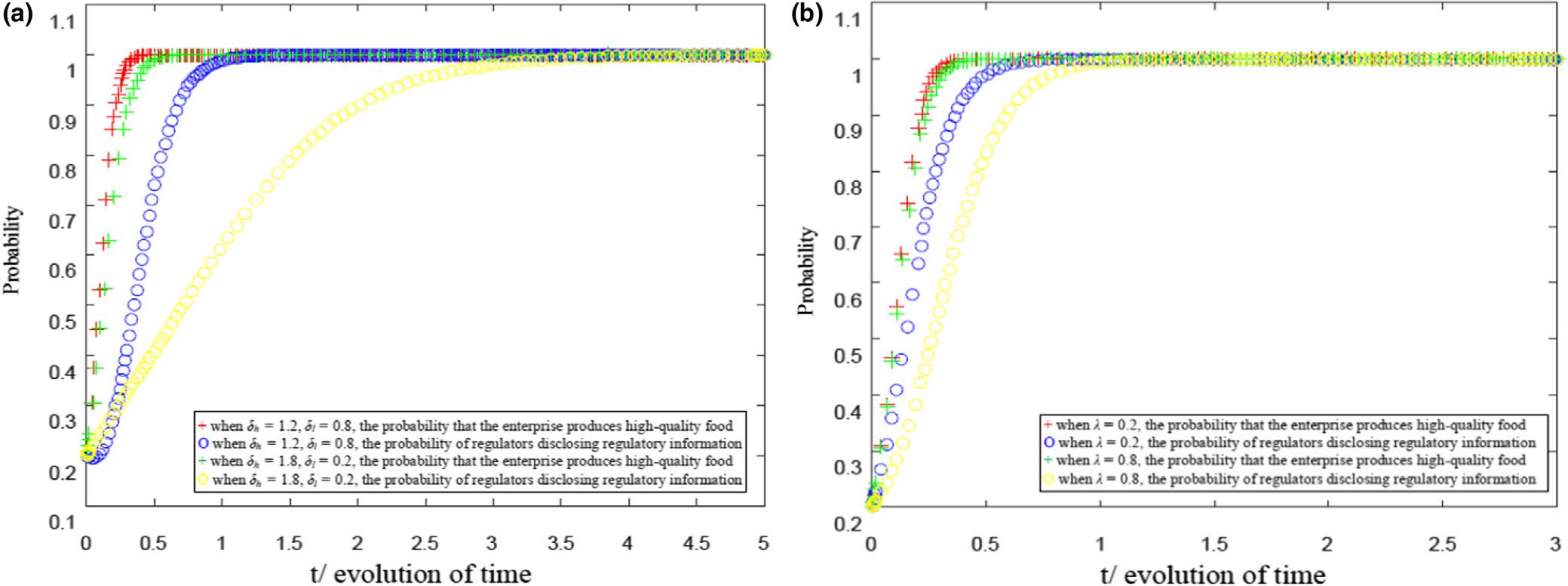
(a) Evolution results of health food safety risk supervision under the constraint (*p_h_*–*c_h_*)*Q*_th_− (*p_l_*−*c_l_*)*Q*_tl_ > 0. (b) Evolution results of health food safety risk supervision under the constraint (*p_h_*−*c_h_*)*Q*_th_− (*p_l_*−*c_l_*)*Q_tl_* > 0

When the condition $\left(p_{h}-c_{h}\right)Q_{\mathrm{th}}-\left(p_{l}-c_{l}\right)Q_{\mathrm{tl}}>0$ is met, MATLAB 2016b is used to simulate and obtain the strategy evolution process of health food enterprises and government regulatory departments, as shown in Figure 1 refer to appendix. Figure 1a compares the impact of consumers’ sensitivity to regulatory information on the evolution speed of strategic choice of both game players. Figure 1b compares the impact of the value of policy burden on the evolution speed of strategic choice of both game players. During the calculation of experimental simulation, the parameter values are set as follows: $p_{h}=3$, $p_{l}=2$, $c_{h}=2$, $c_{l}=1.3$, a=20, b=1, F=10, $C_{b}=5$, and R=10.

Moreover, in Figure 1a, λ=0.5, and in Figure 1b, $\delta_{h}=1.5$ and $\delta_{l}=0.5$. Figure 1a reveals that the higher the consumers’ sensitivity to government regulatory information on health food safety, the faster the stabilization of the strategy of health food enterprises and government regulatory departments with ESS (1, 1). Therefore, the higher the capability of information acquisition and recognition of consumers, the easier the promotion of producing quality health food among health food enterprises and the more able that the government can be urged to disclose health food safety risk supervision information in a timely manner. As a result, the healthy development of the health food market is guaranteed. Figure 1b shows that the higher the policy burden, the slower the government regulatory authorities’ strategy to stabilize the disclosure of health food safety risk regulatory information. Therefore, the higher the policy burden, the less likely it is to promote the government to disclose health food safety risk regulatory information.

### 4.2 Simulation of the evolutionary dynamics of health food safety risk supervision when $\left(p_{h}-c_{h}\right)Q_{\mathrm{th}}-\left[\left(p_{l}-c_{l}\right)Q_{\mathrm{tl}}-F-C_{b}\right]<0$, $\delta_{h}\left(p_{h}-c_{h}\right)Q_{\mathrm{th}}-\left[\delta_{l}\left(p_{l}-c_{l}\right)Q_{\mathrm{tl}}-F\right]>0$, and $\lambda \delta_{l}\left(p_{l}-c_{l}\right)Q_{\mathrm{tl}}-\left[\lambda \left(p_{l}-c_{l}\right)Q_{\mathrm{tl}}+C_{b}-R\right]>0$

**Figure 2:**
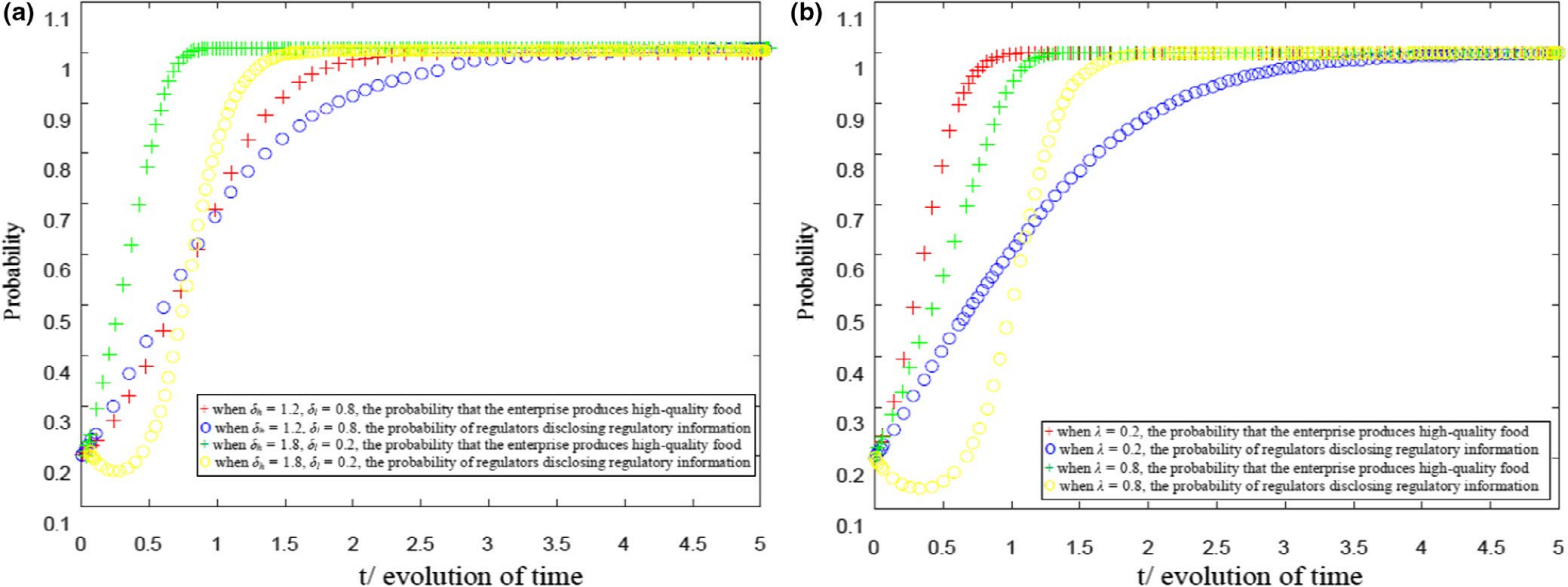
(a) Evolution results of health food safety risk supervision under the constraints (*p_h_*−c_h_)*Q_th_*− [(*p_l_*−*c_l_*)*Q*_tl_−*F*−*C_b_*]<0, *δ_h_* (*p_h_*−c_h_)*Q_th_*− [*δ_h_* (*p_l_*−*c_l_*)*Q_tl_*−*F*] > 0, and *λδ_l_* (*p_l_*−*c_l_*)*Q_tl_*−[*λ*(*p_l_*−*c_l_*)*Q_tl_*+*C_b_*−*R*] > 0. Evolution results of health food safety risk supervision under the constraints (*p_h_*−*c_h_*)*Q_th_*−[(*p_l_*−*c_l_*)*Q_tl_*−*F*−*C_b_*]<0, *δ_h_* (*p_h_*−*c_h_*)*Q_th_*−[*δ_h_* (*p_l_*−*c_l_*)*Q_tl_*−*F*] > 0, and *λδ_l_* (*p_l_*−*c_l_*)*Q_tl_*− [*λ*(*p_l_*−*c_l_*)*Q_tl_*+*C_b_*−*R*]>0.

When conditions $\left(p_{h}-c_{h}\right)Q_{\mathrm{th}}-\left[\left(p_{l}-c_{l}\right)Q_{\mathrm{tl}}-F-C_{b}\right]<0$, $\delta_{h}\left(p_{h}-c_{h}\right)Q_{\mathrm{th}}-\left[\delta_{l}\left(p_{l}-c_{l}\right)Q_{\mathrm{tl}}-F\right]>0$, and $\lambda \delta_{l}\left(p_{l}-c_{l}\right)Q_{\mathrm{tl}}-\left[\lambda \left(p_{l}-c_{l}\right)Q_{\mathrm{tl}}+C_{b}-R\right]>0$ are met, MATLAB 2016b is used to simulate the strategy evolution process of health food enterprises and government regulatory departments, as shown in Figure 2, refer to appendix. Figure 2a compares the impact of consumers’ sensitivity to regulatory information on the evolution speed of the strategic choice of both game players. Figure 2b compares the impact of the value of policy burden on the evolution speed of the strategic choice of both game players. During the calculation of the experimental simulation, the parameter values are set as follows:$p_{h}=5$,$c_{h}=3.5$,$p_{l}=4$,$c_{l}=2$,a=20,b=2,F=5,$C_{b}=5$, and R=10.

Moreover, in Figure 2a, λ=0.5 and in Figure 2b, $\delta_{h}=1.5$ and $\delta_{l}=0.5$. Figure 2a reveals that the higher the consumers’ sensitivity to health food safety information, the faster the evolution of the strategy of health food enterprises and government regulators to ESS (1, 1). That is, with the disclosure of government regulatory information on health food safety, the profitability of health food enterprises producing quality health food can be rapidly improved due to the high sensitivity of consumers to information. As a result, the production of quality health food in health food enterprises is promoted. Similarly, without considering the impact of the policy burden, when the profitability of enterprises producing quality health food can be significantly improved, the earnings of government regulatory departments can also be accordingly improved. Combined with the influence of various factors, such as social credibility, the strategy of regulatory authorities is finally stable in disclosing health food safety regulatory information. Figure 2b shows that, in this case, the higher the policy burden, the faster the stability of government regulatory authorities’ information disclosure strategy with the disclosure of health food safety regulatory information. The reason for this situation is that, due to consumers’ high sensitivity to information, the disclosure of government regulatory information can help urge health food enterprises in producing quality health food to obtain higher benefits than those producing inferior health food.

### 4.3 Simulation of the evolutionary dynamics of health food safety risk supervision when $\left(p_{h}-c_{h}\right)Q_{\mathrm{th}}-\left[\left(p_{l}-c_{l}\right)Q_{\mathrm{tl}}-F-C_{b}\right]<0$, $\delta_{h}\left(p_{h}-c_{h}\right)Q_{\mathrm{th}}-\left[\delta_{l}\left(p_{l}-c_{l}\right)Q_{\mathrm{tl}}-F\right]<0$, and $\lambda \delta_{l}\left(p_{l}-c_{l}\right)Q_{\mathrm{tl}}-\left[\lambda \left(p_{l}-c_{l}\right)Q_{\mathrm{tl}}+C_{b}-R\right]>0$

**Figure 3:**
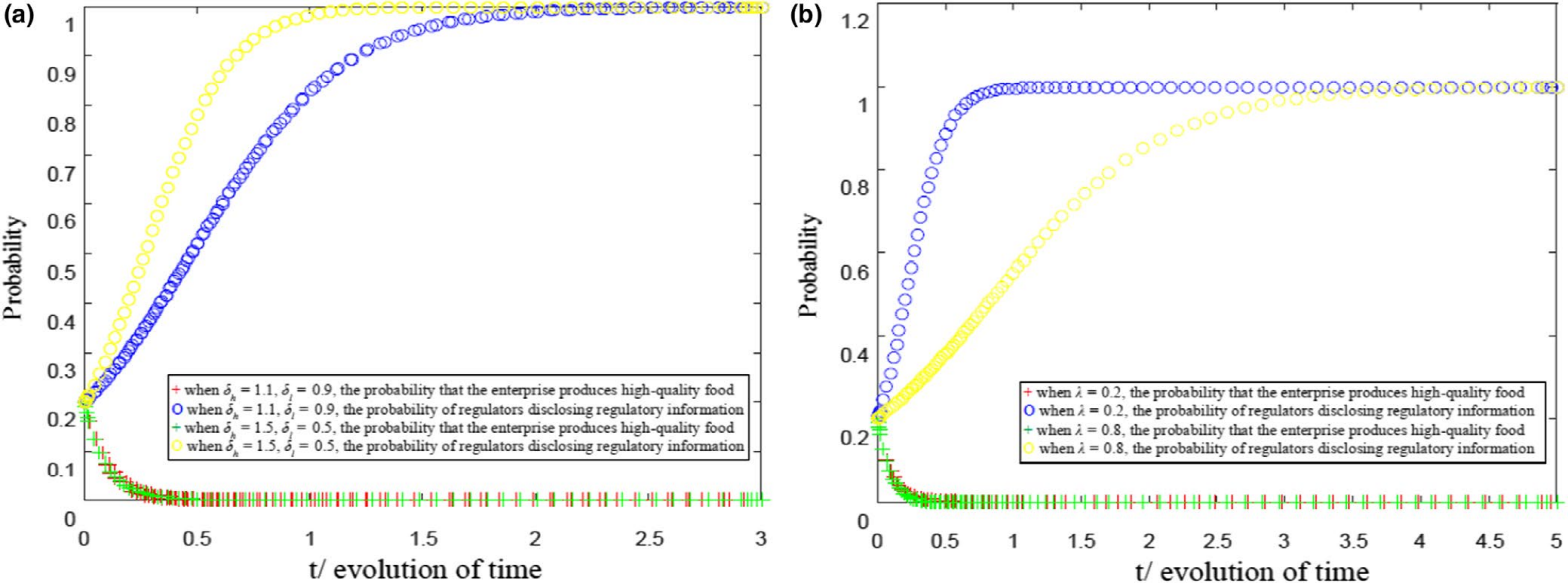
Evolution results of health food safety risk supervision under the constraints (*p_h_* −c_h_)*Q*_th_− [(p_l_−*c_l_*)*Q_tl_*−F‐*C_b_*]<0, *δ_h_* (*p_h_* −*c_h_*)*Q_th_*− [*δ_h_* (*p_l_*−*c_l_*)*Q_tl_*−*F*]<0, and *λδ_l_*(*p_l_*−*c_l_*)*Q_tl_*‐[*λ*(*p_l_*−*c_l_*)*Q_tl_*+*C_b_−R*]>0. (b) Evolution results of health food safety risk supervision under the constraints (*p_h_* −c_h_)Q_th_− [(*p_l_*−*c_l_*)*Q_tl_*−*F*−*C_b_*]<0, *δ_h_* (*p_h_* −*c_h_*)*Q_th_*− [*δ_h_* (p_l_−*c_l_*)*Q_tl_*−F]<0, and *λδ_l_* (*p_l_*−*c_l_*)*Q_tl_*− [*λ*(*p_l_*−*c_l_*)*Q_tl_*+*C_b_−R*]>0

The obtained evolution process of the strategy of health food enterprises and government regulatory departments when $\left(p_{h}-c_{h}\right)Q_{\mathrm{th}}-\left[\left(p_{l}-c_{l}\right)Q_{\mathrm{tl}}-F-C_{b}\right]<0$,$\delta_{h}\left(p_{h}-c_{h}\right)Q_{\mathrm{th}}-\left[\delta_{l}\left(p_{l}-c_{l}\right)Q_{\mathrm{tl}}-F\right]<0$, and $\lambda \delta_{l}\left(p_{l}-c_{l}\right)Q_{\mathrm{tl}}-\left[\lambda \left(p_{l}-c_{l}\right)Q_{\mathrm{tl}}+C_{b}-R\right]>0$ is shown in Figure 3 refer to appendix. The parameter values are set as follows:$p_{h}=5$, $c_{h}=4$, $p_{l}=4$, $c_{l}=1.5$, a=20, b=3, F=5, $C_{b}=5$, and R=7.

Moreover, in Figure 3a, λ=0.5 and in Figure 3b, $\delta_{h}=1.5$ and $\delta_{l}=0.5$. Figure 3a shows that the higher the consumers’ sensitivity to government regulatory information about health food safety, the faster the evolution and stabilization of regulatory authorities’ disclosure of health food safety regulatory information. Similarly, Figure 3b reveals that the higher the policy burden, the faster the stability of information disclosure strategy selection of government regulatory departments with the disclosure of health food safety regulatory information. The reason for the evolution of the disclosure strategy of government regulatory information in Figure 3 is basically consistent with that in Figure 2. The profit gained by collusion cannot compensate for the loss caused by the decline of social credibility.

### 4.4 Simulation of the evolutionary dynamics of health food safety risk supervision when $\left(p_{h}-c_{h}\right)Q_{\mathrm{th}}-\left[\left(p_{l}-c_{l}\right)Q_{\mathrm{tl}}-F-C_{b}\right]<0$, $\delta_{h}\left(p_{h}-c_{h}\right)Q_{\mathrm{th}}-\left[\delta_{l}\left(p_{l}-c_{l}\right)Q_{\mathrm{tl}}-F\right]<0$, and $\lambda \delta_{l}\left(p_{l}-c_{l}\right)Q_{\mathrm{tl}}-\left[\lambda \left(p_{l}-c_{l}\right)Q_{\mathrm{tl}}+C_{b}-R\right]<0$

**Figure 4:**
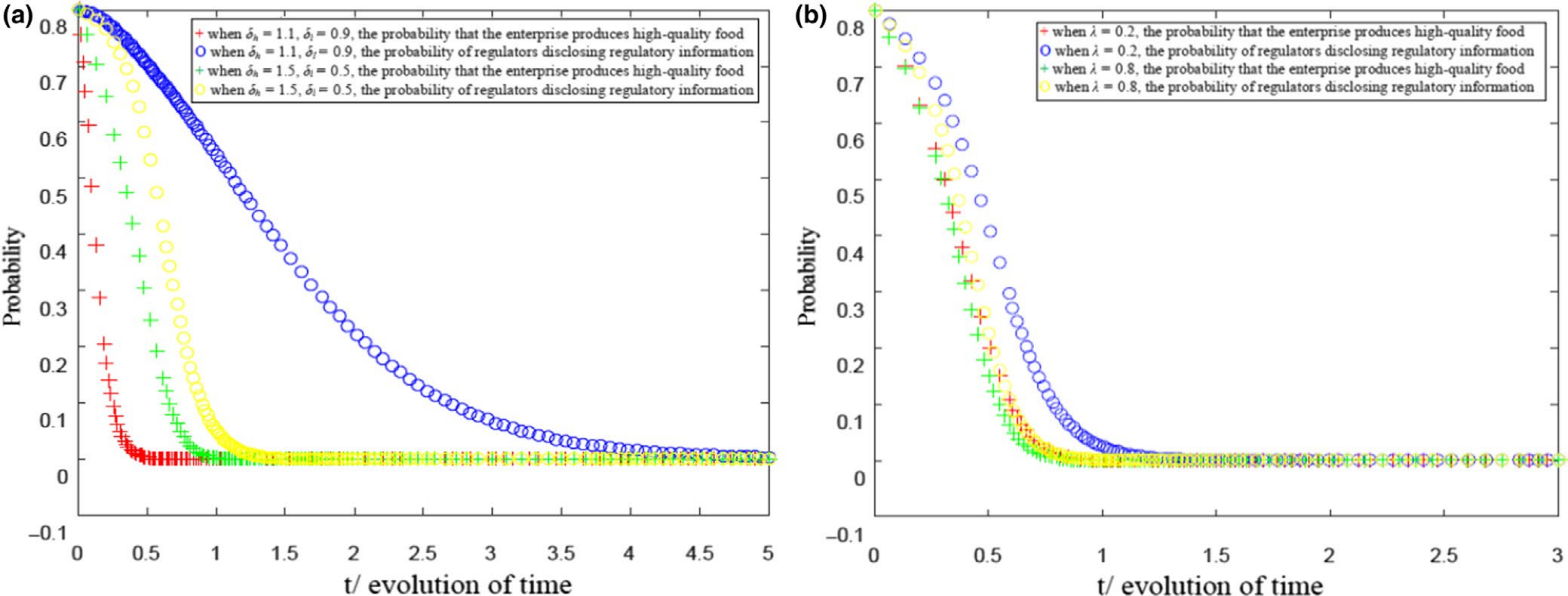
Evolution results of health food safety risk supervision under the constraints (*p_h_* −*c_h_*)*Q_h_*− [(*p_l_*−*c_l_*)*Q_tl_*−*F*−*C_b_*]<0, *δ_h_* (*p_h_* −*c_h_*)*Q_th_*− [*δ_h_*(*p_l_*−*c_l_*)*Q_tl_*−*F*] < 0, and *λδ_l_* (*p_l_*−*cl*)*Q_tl_*− [*λ*(*p_l_*−*c_l_*)*Q_tl_*+*C_b_*−*R*]<0. (b) Evolution results of health food safety risk supervision under the constraints (*p_h_* −*c_h_*)*Q_th_*− [(*p_l_*−*c_l_*)*Q_tl_*−*F*−*C_b_*]<0, *δ_h_* (*p_h_*−*c_h_*)*Q_th_*−[*δ_h_* (*p_l_*−*c_l_*)*Q_tl_*−*F*]<0, and *λδ_l_* (*p_l_*−*c_l_*)*Q_tl_*− [λ(*p_l_*−*c_l_*)*Q_tl_*+*C_b_*−*R*]<0

The obtained evolution process of the strategy of health food enterprises and government regulatory departments when $\left(p_{h}-c_{h}\right)Q_{\mathrm{th}}-\left[\left(p_{l}-c_{l}\right)Q_{\mathrm{tl}}-F-C_{b}\right]<0$, $\delta_{h}\left(p_{h}-c_{h}\right)Q_{\mathrm{th}}-\left[\delta_{l}\left(p_{l}-c_{l}\right)Q_{\mathrm{tl}}-F\right]<0$, and $\lambda \delta_{l}\left(p_{l}-c_{l}\right)Q_{\mathrm{tl}}-\left[\lambda \left(p_{l}-c_{l}\right)Q_{\mathrm{tl}}+C_{b}-R\right]<0$ are shown in Figure 4 refer to appendix. The parameter values are set as follows:$p_{h}=5$, $c_{h}=4$, $p_{l}=4$, $c_{l}=2$, a=20, b=2, F=5, $C_{b}=5$, and R=5.

Moreover, in Figure 4a, λ=0.5 and in Figure 4b, $\delta_{h}=1.5$ and $\delta_{l}=0.5$. Figure 4 shows that when the improvement of consumers’ sensitivity on health food safety, government regulation information cannot easily improve the profit of health food enterprises producing high‐quality health food. Moreover, when the benefits of government collusion with health food enterprises are higher than the loss of public trust, the health food market can be out of control. The production of low‐quality health food is the optimal strategy of health food enterprises, whereas colluding with health food enterprises to secretly report the supervision information about health food safety is the optimal strategy of government supervision departments. In addition, Figure 4a shows that when constraints $\left(p_{h}-c_{h}\right)Q_{\mathrm{th}}-\left[\left(p_{l}-c_{l}\right)Q_{\mathrm{tl}}-F-C_{b}\right]<0$, $\delta_{h}\left(p_{h}-c_{h}\right)Q_{\mathrm{th}}-\left[\delta_{l}\left(p_{l}-c_{l}\right)Q_{\mathrm{tl}}-F\right]<0$, and $\lambda \delta_{l}\left(p_{l}-c_{l}\right)Q_{\mathrm{tl}}-\left[\lambda \left(p_{l}-c_{l}\right)Q_{\mathrm{tl}}+C_{b}-R\right]<0$ are met, the more sensitive consumers are to the regulatory information of health food safety, the faster the evolution of government regulatory authorities’ strategic choice to nondisclose the regulatory information of health food safety. Moreover, Figure 4b shows that as the collusion with health food enterprises can bring high benefits to the government, the higher the policy burden, the faster the evolution of the government's information disclosure strategy to the nondisclosure of regulatory information.

## 5 CONCLUSIONS AND IMPLICATIONS

### 5.1 Research conclusions

The information disclosure strategies of government regulators and the production strategy selection of health food enterprises are dynamic processes of mutual influence. At the same time, the evolution speed of strategies is affected by consumers’ sensitivity to food safety regulatory information and the policy burden of the government. This study considered the government's policy burdens and the degree of consumer response to food safety regulatory information. It aims to build a model of health food enterprise production and operation and government regulatory information disclosure and analyze the evolution process of the government behavior of regulatory information disclosure of health food safety. The theoretical derivation and simulation analysis found that the more sensitive consumers are to the supervision information on health food safety, the more significant the improvement of the profitability of enterprises producing quality health food Thus, objective and comprehensive disclosure of health food safety becomes highly promoted in the government. The following are the conditions for government regulators in voluntarily disclosing health food safety information to provide consumer guidance: (1) When consumers have sufficient ability to identify the quality of health food and when the government can eliminate health food enterprises producing inferior quality health food through market means, that is, $\left(p_{h}-c_{h}\right)Q_{\mathrm{th}}-\left(p_{l}-c_{l}\right)Q_{\mathrm{tl}}>0$; (2) when the initial profitability of health food enterprises in the production of high quality health food is less than that of health food enterprises in the production of inferior health food, but consumers are sensitive to government regulatory information about health food safety and can adjust consumption behaviors according to the regulatory information to effectively improve the profitability of health food enterprises producing high‐quality health food, that is, $\left(p_{h}-c_{h}\right)Q_{\mathrm{th}}-\left[\left(p_{l}-c_{l}\right)Q_{\mathrm{tl}}-F-C_{b}\right]<0$ and $\delta_{h}\left(p_{h}-c_{h}\right)Q_{\mathrm{th}}-\left[\delta_{l}\left(p_{l}-c_{l}\right)Q_{\mathrm{tl}}-F\right]>0$; and (3) when consumer response to regulatory information cannot easily improve the profitability of health food enterprises producing quality health food, and the benefits obtained by regulatory departments from the collusion with health food enterprises cannot make up for the losses caused by the declining social credibility, that is, $\lambda \delta_{l}\left(p_{l}-c_{l}\right)Q_{\mathrm{tl}}-\left[\lambda \left(p_{l}-c_{l}\right)Q_{\mathrm{tl}}+C_{b}-R\right]>0$.

### 5.2 Inspiration and countermeasures

On the basis of the given conclusions of the political and enterprise evolution game theory and the actual situation of the health food market, this study proposes the following countermeasures to improve the mechanism of health food safety supervision and promote a healthy development of the health food market:

1. Publicity and popularization of health food safety knowledge in the whole society should be performed to improve the ability of consumers in identifying the quality of health food. When consumers have a high ability of identifying the quality of health food, for enterprises, the production of quality health food can obtain more profits, thus increasing the earnings of enterprises. At the same time, the probability that government regulators and health food enterprises collude with each other is increased, which can force government regulators to reduce the probability of collusion with health food enterprises.
2. The media and relevant public welfare organizations should actively release and reprint health food safety warning information, reducing consumers’ cost of information searching and improving their sensitivity to information. The improvement of consumers’ sensitivity to health food safety information also improves the profitability of enterprises producing quality health food, thus meeting the constraints $\left(p_{h}-c_{h}\right)Q_{\mathrm{th}}-\left[\left(p_{l}-c_{l}\right)Q_{\mathrm{tl}}-F-C_{b}\right]<0$ and $\delta_{h}\left(p_{h}-c_{h}\right)Q_{\mathrm{th}}-\left[\delta_{l}\left(p_{l}-c_{l}\right)Q_{\mathrm{tl}}-F\right]>0$.
3. When consumers’ sensitivity to information is not high, the policy burden of the government is reduced. When consumers are not sensitive to health food safety supervision information, $\left(p_{h}-c_{h}\right)Q_{\mathrm{th}}-\left[\left(p_{l}-c_{l}\right)Q_{\mathrm{tl}}-F-C_{b}\right]<0$ and $\delta_{h}\left(p_{h}-c_{h}\right)Q_{\mathrm{th}}-\left[\delta_{l}\left(p_{l}-c_{l}\right)Q_{\mathrm{tl}}-F\right]<0$. As a result, the government's policy dependence on health food enterprises can reduce the willingness of supervision departments to collude with health food enterprises, thus urging them to disclose comprehensive and real health food safety supervision information to the whole society.
4. Social credibility must be brought into the performance appraisal of government regulatory departments. Penalties for their loss of social credibility must also be increased. If the regulator's loss of social credibility can be made up by the benefits of collusion, that is, $\lambda \delta_{l}\left(p_{l}-c_{l}\right)Q_{\mathrm{tl}}-\left[\lambda \left(p_{l}-c_{l}\right)Q_{\mathrm{tl}}+C_{b}-R\right]<0$, then the result of the strategy evolution of the regulatory authorities will be the nondisclosure of health food safety regulatory information. As a result, the production of inferior health food becomes the best strategy of health food enterprises.

## CONFLICT OF INTERESTS

The authors declare that they have no competing interests.

## ETHICAL APPROVAL

This study does not involve any human or animal testing.

## References

[fsn31257-bib-0001] Akerlof, G. (1970). The Market for “Lemons”: Quality Uncertainty and the Market Mechanism. Quarterly Journal of Economics, 84(3), 488–500. 10.2307/1879431

[fsn31257-bib-0002] Arthur, P. J. M. (2014). Governing China's food quality through transparency: A review. Food Control, 43, 49–56. 10.1016/j.foodcont.2014.02.034

[fsn31257-bib-0003] Bai, L. I. , Ma, C.‐L. , Yang, Y.‐S. , Zhao, S.‐K. , & Gong, S.‐L. (2007). Implementation of HACCP system in China: A survey of food enterprises involved. Food Control, 18(9), 1108–1112. 10.1016/j.foodcont.2006.07.006

[fsn31257-bib-0004] Chen, T. , Ma, B. , & Wang, J. (2018). SIRS contagion model of food safety risk. Journal of Food Safety, 2, e12410 10.1111/jfs.12410

[fsn31257-bib-0005] Chen, T. , Wang, L. , & Wang, J. (2017). Transparent assessment of the supervision information in China’s food safety: A Fuzzy‐ANP comprehensive evaluation method. Journal of Food Quality, 2017(9), 1–14. 10.1155/2017/4340869

[fsn31257-bib-0006] Chira, A. , Chira, L. , & Delian, E. (2011). Studies regarding the implementation and certification of food safety management system in the Romanian companies. Bulletin of university of agricultural sciences and veterinary medicine Cluj‐Napoca. Agriculture, 296(1), 63–69.

[fsn31257-bib-0007] Darby, M. R. , & Karni, E. (1973). Free competition and the optimal amount of fraud. The Journal of Law and Economics, 16(1), 67–88. 10.1086/466756

[fsn31257-bib-0008] Dreyer, M. , Renn, O. , Cope, S. , & Frewer, L. J. (2010). Including social impact assessment in food safety governance. Food Control, 21(12), 1620–1628. 10.1016/j.foodcont.2009.05.007

[fsn31257-bib-0009] Dulleck, U. , Kerschbamer, R. , & Sutter, M. (2009). The economics of credence goods: An experiment on the role of liability, verifiability, reputation, and competition. American Economic Review, 101(2), 526–555. 10.1257/aer.101.2.526

[fsn31257-bib-0010] Fan, H. , Ye, Z. , Zhao, W. , Tian, H. , Qi, Y. , & Busch, L. (2009). Agriculture and food quality and safety certification agencies in four Chinese cities. Food Control, 20(7), 627–630. 10.1016/j.foodcont.2008.09.013

[fsn31257-bib-0011] Fernando, Y. , Ng, H. H. , & Walters, T. (2015). Regulatory incentives as a moderator of determinants for the adoption of Malaysian food safety system. British Food Journal, 117(4), 1336–1353. 10.1108/BFJ-03-2014-0129

[fsn31257-bib-0012] Firdaus, S. A. , Son, R. , Mohhiddin, O. et al. (2015). Food court hygiene assessment and food safety knowledge, attitudes and practices of food handlers in Putrajaya. International Food Research Journal, 22(5), 1843–1854.

[fsn31257-bib-0013] Franco, M. , Diez Roux, A. V. , Glass, T. A. , Caballero, B. , & Brancati, F. L. (2008). Neighborhood characteristics and availability of healthy foods in Baltimore. American Journal of Preventive Medicine, 35(6), 561–567. 10.1016/j.amepre.2008.07.003 18842389 PMC4348113

[fsn31257-bib-0014] Helm, D. (2006). Regulatory reform, capture, and the regulatory burden. Oxford Review of Economic Policy, 22(2), 169–185. 10.1093/oxrep/grj011

[fsn31257-bib-0015] Kleve, P. , & Mulder, R. D. (2008). Privacy protection and the right to information: In search of a new balance. Computer Law & Security Review, 24(3), 223–232. 10.1016/j.clsr.2008.03.008

[fsn31257-bib-0016] König, A. (2010). Compatibility of the SAFE FOODS Risk Analysis Framework with the legal and institutional settings of the EU and the WTO. Food Control, 21(12), 1638–1652. 10.1016/j.foodcont.2009.11.018

[fsn31257-bib-0017] Li, T. , Bernard, J. C. , Johnston, Z. A. , Messer, K. D. , & Kaiser, H. M. (2017). Consumer preferences before and after a food safety scare: An experimental analysis of the 2010 egg recall. Food Policy, 66, 25–34. 10.1016/j.foodpol.2016.11.008

[fsn31257-bib-0018] Liu, S. , Chen, S. , Su, Y. et al. (2013). Soil erosion control in the karst mountain area of north Guangdong province from the perspective of farmers behavior‐case study in shaping township of Shaoguan City. Subtropical Soil & Water Conservation, 55(25), 102231.

[fsn31257-bib-0019] Martinez, M. G. , Verbruggen, P. , & Fearne, A. (2013). Risk‐based approaches to food safety regulation: What role for co‐regulation? Journal of Risk Research, 16(9), 1101–1121. 10.1080/13669877.2012.743157

[fsn31257-bib-0020] Milios, K. , Zoiopoulos, P. , Pantouvakis, A. , Mataragas, M. , & Drosinos, E. (2013). Techno‐managerial factors related to food safety management system in food businesses. British Food Journal, 115(9), 1381–1399. 10.1108/BFJ-11-2011-0284

[fsn31257-bib-0021] Ni, H. G. , & Zeng, H. (2009). Law enforcement is key to China's food safety. Environmental Pollution, 157(7), 1990–1992. 10.1016/j.envpol.2009.02.002 19264385

[fsn31257-bib-0022] Nyokabi, S. , Birner, R. , Bett, B. , Isuyi, L. , Grace, D. , Güttler, D. , & Lindahl, J. (2017). Informal value chain actors' knowledge and perceptions about zoonotic diseases and biosecurity in Kenya and the importance for food safety and public health. Tropical Animal Health & Production, 50(3), 509–518. 10.1007/s11250-017-1460-z 29130123 PMC5818561

[fsn31257-bib-0023] Powell, D. A. , Erdozain, S. , Dodd, C. , Costa, R. , Morley, K. , & Chapman, B. J. (2013). Audits and inspections are never enough: A critique to enhance food safety. Food Control, 30(2), 686–691. 10.1016/j.foodcont.2012.07.044

[fsn31257-bib-0024] Pu, X. J. , Lu, L. , & Han, X. H. (2014) Certification of credence goods with consideration of consumers' learning ability[C]// International Conference on Management Science & Engineering. IEEE 596‐603.

[fsn31257-bib-0025] Rosenau, J. N. , & Czempiel, E. (1992). Governance without government: Order and change in world politics. New York, NY: Cambridge University Press.

[fsn31257-bib-0026] Sawada, Y. , Kondo, K. , & Ito, M. (2009). Comparison of the information disclosure systems for the nursing facilities in Japan and the U.S. Japanese Journal of Social Welfare, 50, 95–109.

[fsn31257-bib-0027] Story, M. , Kaphingst, K. M. , Robinson‐O'Brien, R. , & Glanz, K. (2008). Creating healthy food and eating environments: Policy and environmental approaches. Annual Review of Public Health, 29(1), 253–272. 10.1146/annurev.publhealth.29.020907.090926 18031223

[fsn31257-bib-0028] Teixeira, S. , & Sampaio, P. (2013). Food safety management system implementation and certification: Survey results. Total Quality Management & Business Excellence, 24(3–4), 275–293. 10.1080/14783363.2012.669556

[fsn31257-bib-0029] Umehara, E. , & Ohta, T. (2009). Using game theory to investigate risk information disclosure by government agencies and satisfying the public—the role of the guardian agent. IEEE Transactions on Systems, Man, and Cybernetics ‐ Part A: Systems and Humans, 39(2), 321–330. 10.1109/TSMCA.2008.2010796

[fsn31257-bib-0030] Van Boxstael, S. , Habib, I. , Jacxsens, L. , De Vocht, M. , Baert, L. , Van De Perre, E. , … Uyttendaele, M. (2013). Food safety issues in fresh produce: Bacterial pathogens, viruses and; pesticide residues indicated as major concerns by stakeholders in the; fresh produce chain. Food Control, 32(1), 190–197. 10.1016/j.foodcont.2012.11.038

[fsn31257-bib-0031] Waldman, K. B. , & Kerr, J. M. (2018). Does safety information influence consumers’ preferences for controversial food products? Food Quality & Preference, 64 10.1016/j.foodqual.2017.10.013

[fsn31257-bib-0032] Wang, J. (2015). Weighing the public interest in the disclosure of government information. Social Sciences in China, 36(3), 37–55. 10.1080/02529203.2015.1062228

[fsn31257-bib-0033] Wang, J. , & Chen, T. (2016). The spread model of food safety risk under the supply‐demand disturbance. Springerplus, 5(1), 1765 10.1186/s40064-016-2881-2 27795907 PMC5056927

[fsn31257-bib-0034] Wang, J. , Chen, T. , & Wang, J. (2015). Research on cooperation strategy of enterprises’ quality and safety in food supply chain. Discrete Dynamics in Nature and Society, 3, 1–15. 10.1155/2015/301245

[fsn31257-bib-0035] Wang, Y. , Liu, S. , Du, J. et al. (2014). Contagion effects vs. competitive effects in credence goods markets: theory and event study on china's food markets. Economic Research Journal, 2, 141–154.

[fsn31257-bib-0036] Wilson, S. , Chapman, B. , & Powell, D. (2011). Understanding Food Safety Information Needs: Using a National Information Service as a Research Tool. Food Protection Trends, 31(7), 437–445.

[fsn31257-bib-0037] Wu, Y. (2012). Information Infrastructure, Reputation Mechanism and the Optimization of Law Enforcement: A New View of Food Safety Management. Social Sciences in China, 06, 115–133.

[fsn31257-bib-0038] Zimper, A. , & Hassan, S. (2012). Can industry regulators learn collusion structures from information‐efficient asset markets? Economics Letters, 116(1), 1–4. 10.1016/j.econlet.2012.01.002

